# Perceptions and experiences of healthcare professionals of implementing the Organ Donation (Deemed Consent) Act in England during the Covid-19 pandemic

**DOI:** 10.1186/s12913-025-12224-8

**Published:** 2025-01-31

**Authors:** Mustafa Al-Haboubi, Leah McLaughlin, Lorraine Williams, Jane Noyes, Stephen O’Neill, Paul Boadu, Jennifer Bostock, Nicholas Mays

**Affiliations:** 1 Policy Innovation and Evaluation Research Unit, Department of Health Services Research and Policy, London School of Hygiene & Tropical Medicine, London, UK; 2 School of Medical and Health Sciences, Bangor University, Bangor, UK

**Keywords:** Deemed consent, Organ donation, Implementation

## Abstract

**Context:**

In May 2020 during the COVID-19 pandemic, England implemented a ‘soft’ opt-out system of consent to deceased organ donation. As part of a wider evaluation, this analysis focused on the perceptions of health care professionals, specifically their experiences of implementation.

**Methods:**

Mixed-methods study informed by Normalisation Process Theory, based on two national surveys of health care professionals and interviews, observations and document analysis, across two case study sites. Routine NHS Blood and Transplant’s audit data provided context.

**Findings:**

70 interviews with 59 staff and 244 first and 738 second surveys. COVID-19 affected every aspect of implementation. Although supportive in principle, many staff were unconvinced that legislative changes alone would increase consent rates. Many staff were redeployed or left their jobs. As a result, staff were not able to work collectively as intended for implementation. Staff received routine donor audit data suggesting the law was yet to make a difference to consent rates, reducing their enthusiasm and commitment.

**Conclusions:**

Implementation could have been more impactful if delayed. The National Health Service needs to reprioritise organ donation and relaunch the implementation plan in the post-pandemic period, though it is unlikely the changes will bring about a significant increase in consent rates.

**Supplementary Information:**

The online version contains supplementary material available at 10.1186/s12913-025-12224-8.

## Background

Human organ transplantation aims to improve and prolong the life of the recipient. In 2021, it was estimated that over 144,000 organs were transplanted world-wide [1]. In the United Kingdom (UK), approximately 4,600 transplants were performed in 2022/2023. However, it was also estimated that around 7,000 patients were on waiting lists, with over 430 dying during that period [2]. Consent to deceased organ donation is regarded as one of the main barriers to making more organs available for transplant in the UK [3].

### The policy context

Although there is no definitive evidence that opt-out systems are any more effective at raising organ donation consent rates than opt-in systems [4, 5], between 2015 and 2023 each of the devolved nations of the UK responsible for the National Health Service (NHS) moved to a ‘soft’ opt-out system of consent to deceased organ donation [6]. These are referred to as ‘soft’ opt-out systems because the family of the deceased continue to be involved in the organ donation discussion and can also potentially override the deceased person’s donation decision made in life. Wales was the first UK devolved nation to implement a ‘soft’ opt-out system in 2015, which has yet to demonstrate significantly increased consent rates compared to the previous opt-in system [7].

In May 2020, in the early stages of the COVID-19 pandemic, when health services were required to quickly reconfigure to meet the challenge of caring for thousands of critically sick patients requiring high dependency and intensive care, England implemented its ‘soft’ opt-out system. Under the new system, all adults aged over 18 years were considered to have consented to organ donation (deemed consent), unless they had indicated that they did not want to be a donor during their lifetime by registering an opt-out decision on the Organ Donor Register, by informing their family members, or were in one of the excluded categories. However rather than replace the opt-in system, the new system added a deemed consent pathway. In addition only certain organs, tissues and their use were covered by the ‘soft’ opt-out system [8]. Excluded organs (e.g. the brain), tissues (e.g. the trachea) and their use still required family or another type of consent (e.g. first person) [9].

The law change was intended to be supplemented by a set of initiatives to increase the chances of a successful implementation. These included media campaigns to raise awareness about the change; expansion of the workforce responsible for obtaining support for organ donation from family members; and additional targeted training of NHS staff (See Appendix 1 for the full list of the intended organ donation implementation support package). This package was not delivered as planned, mostly as a result of the COVID-19 Pandemic which coincided with the date when the law change came into effect.

### The UK organ donation system

NHS Blood and Transplantation (NHSBT) is a Special Health Authority [10] that provides blood and transplantation services across the UK [11]. Special Health Authorities deliver services on a national, rather than local level and are created by the Secretary of State through secondary legislation. The deceased organ donation system comprises services in 12 regions, with nine covering England [12]. Any patient with a severe brain injury or a patient where decisions are being made to withdraw treatment is considered a potential donor and should be referred as soon as possible via a national referral telephone number [13]. If the patient is identified as a potential organ donor, a Specialist Requester (SR) or Specialist Nurses in Organ Donation (SNOD) will be mobilised to speak to the family about organ donation and establish the mode of consent. This process has over time become increasingly specialised with the implementation of the SR role and includes bespoke training with a focus on communication [14].

### The study

This analysis was part of a wider evaluation examining the impact(s) of the changes on the whole organ donation system including the public and families who were approached about organ donation after their relative had died [15]. Our objectives were to explore awareness of the law change and the experiences of health care professionals (including their systems of support) who are directly (e.g. NHSBT) and indirectly (e.g. Intensive care, accident and emergency) tasked with delivering organ donation services in England.

We were also interested in exploring the impact of the COVID-19 pandemic on the NHS and organ donation system, which coincided with implementation of the soft opt-out legislation.

## Methods

### Design

We used a mixed-methods convergent analysis design [16]. We sought to capture the views and experiences of an extensive range of respondents (including NHSBT staff, Clinical Leads in Organ Donation (CLODs), emergency care unit staff, operating theatre staff and adult intensive care unit staff) with a widely distributed survey. In parallel, we aimed to gain in-depth understanding of the views of health care professionals tasked with the implementation of the law change, using semi-structured interviews with purposively sampled staff in two purposively selected NHSBT regions, based on organ donation activity, geographic coverage, consent rates and higher than average ethnic minority populations.

The theoretical framework underpinning our exploration of implementation processes and staff perceptions and experiences was Normalisation Process Theory (NPT) [17]. This is a widely used framework for understanding the factors influencing the implementation of policy or service change in the health care sector in terms of the degree to which the change becomes “normalised” by staff. NPT investigates the levels of coherence (sense making); cognitive participation (relational work); collective action (operational work); and reflexive work (appraisal work) involved in the implementation process. NPT guided the survey questions and topic guides for interview, analysis and data integration.

### Data collection tools

The two surveys were designed for the study to collect information about staff awareness and understanding of the law change; degree of support for the change in the law; reasons for supporting/ not supporting it; and the impact of COVID-19. The second survey additionally collected information about the implementation of the changes; their perceived impact in general and on organ donation rates in minority ethnic groups and faith groups; ways of addressing concerns of families whose involvement did not lead to organ donation; and views on NHSBT’s key performance indicators (see Appendix 2). Similarly, the topic guides for the interviews were devised to explore perceptions and experiences, perceived impact, changes over time and COVID-19 considerations. The two surveys and topic guides were shared with key stakeholders (including the study’s advisory group) and the study’s Patient and Public Involvement and Engagement (PPIE) representative (JB) to ensure they captured relevant information.

We took a number of steps to maximise the validity, reliability and rigour of the data we were collecting, and the subsequent analyses. To minimise the risk of social desirability bias of responses to our surveys, we emphasised in our information sheet that responses would be anonymised in reports. We also piloted the survey questions with a number of individuals with similar characteristics to those completing the survey to ensure that the question wording was unambiguous, neutral, and in the case of closed-ended questions, that we had categorical options to select from. We randomised response options where appropriate to minimise primacy bias. We agreed beforehand the analysis plan for the two surveys and the qualitative research. We used the four-dimension criteria (credibility, dependability, confirmability and transferability) as qualitative markers of rigour throughout [18]. Detailed fieldnotes were often read out to the team who were then able to share their expertise and perspectives to help further contextualize data. We also presented emerging findings at meetings attended by our advisory group and wider stakeholders to check whether they found our interpretation of the data credible.

## Sampling

### Surveys

Two surveys were conducted with NHS and NHSBT staff in England (23 August 2021- 10 January 2022 and 14 November 2022- 28 January 2023). This enabled us to observe changes in views over time. In recognition of the expected low response to surveys conducted at a time of high demand on healthcare workers, we sought to invite all workers we could reach with our survey without aiming for a specific sample size. The surveys were disseminated using the online survey platform Qualtrics XM. Invitations to the survey were disseminated using a combination of direct invitations sent by the Legislation Project Lead at NHSBT to NHSBT staff (nurses and managers) and CLODs physicians, alongside cascading invitations through various professional networks and encouraging completion through the British Association of Critical Care Nurses (BACCN) newsletter which has the widest reach of critical care nurses in the UK. Respondents to the first survey were asked to provide an e-mail address for the second one; those who did so, received a direct invitation to complete the second survey. To incentivise the completion of the second survey (in light of the low response to the first survey), we offered a £5 Amazon voucher to the first 500 respondents who completed the second survey.

#### Interviews

Two NHSBT regions were purposively selected as sites for the staff interviews. London was selected for its high(er) numbers of potential organ donors, ethnically diverse population, and concentration of large acute hospitals. The North West England site was selected as it covers north England, has higher than average numbers of ethnic minority and under-represented groups, and covers a wide geographic area. Within each region two NHS Trusts were selected based on factors such as high and low performance regarding organ donation consent rates, NHSBT classification of level 1 and level 2 centres for a high enough level of organ donation activity to examine in more depth the interactions, processes, and activities between NHS and NHSBT staff.

We aimed to undertake two rounds of interviews with a minimum of 20 interviews across each site and follow-up 12-18 months into implementation. However, since the changes were not implemented as planned, it soon became clear that there was no value in continuing the follow-up interviews. Interviews were undertaken by two experienced female researchers (LM & LW), mostly virtual (Teams, Zoom) with a small number face-to- face interviews. LM undertook interviews across both sites and had previously worked with some participants on a similar evaluation in Wales. In each NHS Trust, we identified participants through purposive and snowball sampling and continued our interviews until the point of data saturation (where interviews were not yielding new insights or themes). This included healthcare professionals working directly in organ donation, such as SNODs, SRs and CLODs, and indirectly, e.g., those working in Intensive Care Units (ICU) and Emergency Departments (ED). Key NHSBT personnel, including regional and team managers were contacted directly to identify potential participants to recruit. Each Trust’s CLOD acted as a lead Principal Investigator (PI) to help identify and recruit NHS personnel working within the targeted specialities, namely intensive care units and emergency departments, as well as from other linked clinical areas, e.g., Stroke units. We sent an ‘invitation to participate email’ to the identified individual, along with a participant information sheet.

### Analysis

#### Surveys

Survey responses were mainly analysed and presented as the number of responses (and percentages) by professional group. The small number of responses from some professional groups (especially in the first survey) prevented us from testing the statistical significance of differences in responses. Open-ended narrative responses were analysed alongside qualitative interview data.

#### Interviews

Interviews were transcribed verbatim, coded against NPT [17] and analysed in NVivo 12 using the Framework approach [19, 20]. Familiarisation with the data was achieved by researchers (LM and LW) reading and re-reading transcripts and accompanying audio recordings, and by annotating and making notes and memos on initial thoughts. Visual maps of the NPT constructs aided the analysis. Summary findings were also coded as broadly “positive”, “negative” or “no difference” against NPT in terms of respondent responses to the changes in organ donation system processes, practices and perceived impacts.

### Data integration

The research team met to discuss, refine and agree the analytical coding framework (see Appendix 3) where NPT constructs were interpreted and mapped for both interview and survey data. We analysed the quantitative and qualitative data separately, and then brought the initial findings together into a narrative using NPT as the organising framework to present the findings.

### Reflexivity

The research team consisted of academics and lay representatives with various experience in health, social and policy research and organ donation. Two members of the team (LM and JN) were involved in a similar evaluation of the changes to the Welsh law on organ donation [5]. In Wales, while donation rates were observed to increase in some areas [21], none of these observations could be attributed directly to the law change [5]. We sought to minimise the risk of the views of the researchers influencing the qualitative data collection and interpretation by agreeing the topic guide for the interviews beforehand and conducting joint analysis meetings. The inclusion of a lay member on the team aided objectivity and was used as a ‘bias check’ throughout.

### Stakeholder engagement and Patient and Public Involvement and Engagement (PPIE)

Researchers attended meetings and training events, organ donation committee meetings, NHSBT team meetings including those allocated to Specialist Requesters, SNODs and management and reviews of documents and processes. We recruited a lay member as a core member of the research team, who has experience of organ donation as a carer of a person who became a donor. Our advisory group was made up of expert and lay members including donor families who had input into the data collection, interpretation and validation of results.

### Findings

We recruited 244 NHS and NHSBT staff to the first survey and 738 to the second (see Table 1 for breakdown of survey responses by professional group). It was not possible to calculate the response rate to the surveys, since invitations were disseminated using newsletters to professional groups whose size cannot be measured accurately to provide a denominator for the response rate estimates. A total of 70 interviews representing 59 staff were completed across the two sites (see Table 2 for details).

**Table 1:** Survey responses, by professional group

**Professional group**	**First survey**		**Second survey**	
	**Number of responses**	**% contribution to overall sample**	**Number of responses**	**% contribution to overall sample**
NHSBT staff	105	43%	156	21%
Adult intensive care unit staff	51	21%	413	56%
Clinical Lead in organ donation	44	18%	70	9%
Operating theatre staff	14	6%	24	3%
Emergency care unit staff	6	2%	42	6%
Other	22	9%	33	4%
Total	**242**	**100%**	**738**	**100%**

**Table 2:** Characteristics of interview participants from the two sites

**Site**	**Number**	**Site**	**Number**
**London**		**North-West**	
**NHS staff** including, CLODs, R-CLODs, Specialist consultants, ITU management & nurses, ED and A&E nurses.	N=13* *N=2 second round*	**NHS staff** including, CLODs, R-CLODs, specialist consultants, TRODs, Bereavement care support staff, ITU management and nurses, ED and A&E management and nurses, Link nurses	N=16* *N=5 second round*
**NHSBT staff** including, SNODs, SRs, PDS, and ODC committee members and chairs	N=11* *N=1 second round*	**NHSBT staff** including, SNODs, SRs, Managers, PDS, Tissue services and ODC committee members and chairs	N=14* *N=8 second round*
**Total**	**27 interviews with 24 staff**	**Total**	**43 interviews with 35 staff**

*CLOD *Clinical Leads Organ Donation, *R-CLODs *Regional Clinical Leads Organ Donation, *TROD* Trainee Representative Organ Donation, *ITU* Intensive Treatment Unit, *SNOD* Specialist Nurse Organ Donation, *SR *Specialist Requester, *ODC *Organ Donation Committee, *PDS *Practice Development Specialists, *ED* Emergency Department, *A&E* Accident and Emergency

We report findings using the four NPT constructs (coherence; cognitive participation; collective action and reflexive monitoring). For each construct, an overview is presented from the survey data and then more detailed perspectives from the qualitative data are presented by professional group: NHS, NHSBT or both.

### Coherence: making sense of the law in relation to practice

**Fig. 1:**
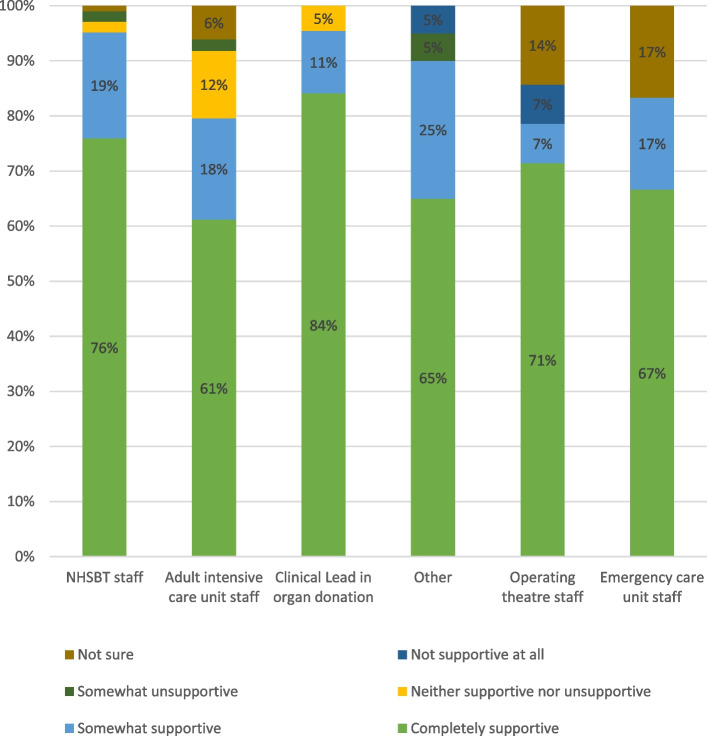
Support for the organ donation law change (first survey)

**Fig. 2:**
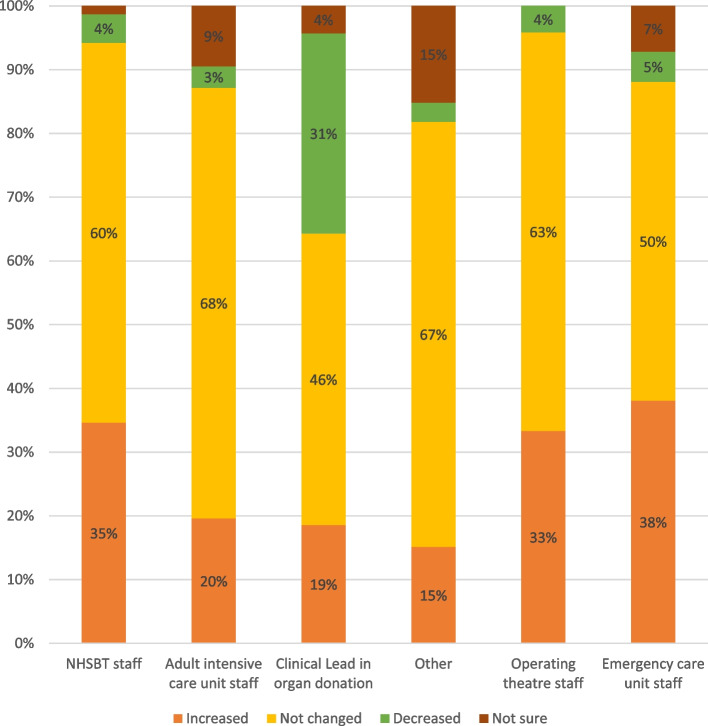
Changes in support for deemed consent since the introduction of the change to the law on organ donation in England in May 2020 (Second survey)

Support for the law change in general was high in the first survey (see Figure 1). The workforce seemed to become more aware of the changes over time (see Figure 2). In general, the more remote from direct involvement in deceased organ donation, the less staff felt that the law change had any relevance for them or impact on their practice. There were more nuanced reflections when we probed deeper in our qualitative research and analysed survey questions with free-text responses, below.

#### NHS staff

Most intensivists took the position that the changes were “*ethically morally, culturally the right thing to do*” (Clinician, interview) but could see no obvious changes to their day-to-day activities. Some were optimistic that the changes might give greater priority to organ donation within intensive care – as something that is delivered as an integrated part of the service – but were cautious about whether the law change would assist with any specific local issues such as bed capacity, staff turnover and burnout. Most preferred to focus and build on what individuals were already doing irrespective of the law change, such as promoting the stories of donors who went on to save multiple (young) lives, excellent examples of multi-disciplinary team working, community engagement and general organ donation promotion.

Many clinicians felt a law change overlaid on the complexity of death and dying conversations, which are ultimately emotional (not rational), would make almost no difference to their practice or consent rates. The ‘softness’ of the legislation was both regarded as ‘good enough’ i.e. at the limit of what is currently likely to be acceptable in the UK, but a cause of frustration especially when trying to explain to colleagues what in fact had changed or what they might need to consider doing differently, if anything. Others commented that the organ donation service might now potentially have put itself in a weaker position by relying on the fact that people have not opted out instead of people having to opt in. They judged that opt in still remains easier to talk about and to promote to families and colleagues especially when organ donation is infrequent. Overall NHS staff in London were more concerned with staffing issues (than the law change), some not currently having a link nurse or even seen a SNOD since before the pandemic.

#### NHSBT staff

Some SNODs/SRs were initially concerned that the law change would take away the family’s capacity to ‘gift’ (as decisions are given to individuals to make while they are alive) and the greater emphasis in performance assessment being given to consent rates. Many explained how the nature of their conversation with families had changed, but that this had evolved over time (especially in the Northwest as many SNODs/SRs had been in post for longer). Most still found talking about the benefits of organ donation easier and preferable than discussing the law. Some were interested to see what the law change would do to their individual consent rates, and a few suggested that the changes in the ways data are collected and presented now would give a more accurate picture of the complexities SNODs/SRs encounter on a daily basis.

#### NHS and NHSBT staff

Within NHSBT and NHS staff involved in organ donation there was overall disappointment that the changes did nothing to stop families overriding the decisions of people who had opted in and many wondered what proportion of “new deemed cases” would have said “yes” anyway under the old system, making measuring change very challenging:

*“How many true deemed, I don’t know. Our conversations have changed, but you’re kind of largely just reinforcing with the family what they were already going to do …”(SR/SNOD interview)*

Survey and interview data reflected similar tensions, especially how, when and where the law would be used to influence families’ behaviours, and this was often couched in terms of concerns about the public’s awareness and understanding:

*“It seems a bit unethical because a line could be crossed and a non-consenting donor who hadn't opted out would be assumed as a donor.” (Second staff survey, Adult intensive care unit staff)*

“*Being required to mention the law and that consent is deemed even though relatives completely support donation makes them feel like something is being taken away from them. We have to deem consent when in fact it is being given*.” (first staff survey, NHSBT staff)

“*SNODs are also discouraged from using the word ‘law’ in family conversations. However, all of the media campaigns use the word ‘law’, and by using the phrase ‘the legislation has changed’ we are making the assumption that everyone understands what we are referring to.” (First staff survey, NHSBT staff)*

“*Although some families are aware of the law change, many still aren’t and so it can become quite difficult when you bring in the notion of a ‘change in law’ when families themselves aren’t even aware and so feel their loved one may not have been either, so have been unable to opt out.” (First staff survey, NHSBT staff)*

Cognitive participation; relational work to building, sustaining, reproducing and transforming practice to implement the new system

NHSBT staff who responded to the first survey felt that they had received sufficient training, personal development and support in carrying out tasks in relation to the law. For wider NHS staff, there was variation between professional groups in terms of training received (see Tables 3, 4 and 5 for details).

**Table 3:** Percentage received/ haven’t received in-house training/ professional development on donation law (First survey)

Professional group	**Received training**	**Haven't received training**	**Can't remember**	**Total number**
NHSBT staff	100%	0%	0%	**104**
Adult intensive care unit staff	44%	44%	13%	**48**
Clinical Lead in organ donation	33%	64%	3%	**36**
Operating theatre staff	38%	54%	8%	**13**
Emergency care unit staff	17%	83%	0%	**6**
Other	41%	53%	6%	**17**
**Total**	**150**	**65**	**9**	**224**

**Table 4:** Whether received training/ professional development on donation law from professional body/ association (First survey*)*

**Professional group**	**Percentage within professional group**			**Total number**
	**Received training**	**Haven’t received training**	**Can’t remember**	
NHSBT staff	31%	63%	7%	**88**
Adult intensive care unit staff	42%	53%	5%	**43**
Clinical Lead in organ donation	43%	49%	8%	**37**
Operating theatre staff	31%	62%	8%	**13**
Emergency care unit staff	0%	100%	0%	**6**
Other	38%	63%	0%	**16**
**Total**	**71**	**120**	**12**	**203**

**Table 5:** Whether received training/ professional development on donation law from NHSBT (First survey)

**Professional group**	**Percentage within professional group**			**Total number**
	**Received Training**	**Haven’t received training**	**Can’t remember**	
Adult intensive care unit staff	53%	35%	12%	**43**
Clinical Lead in organ donation	93%	2%	5%	**43**
Operating theatre staff	23%	62%	15%	**13**
Emergency care unit staff	17%	83%	0%	**6**
Other	63%	32%	5%	**19**
**Total**	**79**	**35**	**10**	**124**

Nonetheless, nearly two thirds (69%) of non-NHSBT staff felt that they were adequately prepared for the law change (see Table 6) and so was their organisation (63%) (see Table 7). Interviews and free-text responses however, revealed challenges from the NHS’s and NHSBT’s perspectives.

**Table 6:** Extent to which non-NHST staff agree with the statement “I feel that I was adequately prepared for the change in the law on organ donation”, by professional group (First survey)

**Professional group**	**Percentage of respondents**					**Total number**
	**Strongly agree**	**Agree**	**Disagree**	**Strongly disagree**	**Not sure**	
Adult intensive care unit staff	10%	43%	27%	8%	12%	**49**
Clinical Lead in organ donation	41%	55%	5%	0%	0%	**44**
Operating theatre staff	7%	36%	14%	14%	29%	**14**
Emergency care unit staff	0%	17%	33%	33%	17%	**6**
Other	25%	60%	0%	5%	10%	**20**
**Total**	**29**	**63**	**19**	**9**	**13**	**133**

**Table 7:** Extent to which non-NHST staff agree with the statement “I feel that my organisation was adequately prepared for the change in the law on organ donation”, by professional group (First survey)

**Professional group**	**Percentage of respondents**					**Total number**
	**Strongly agree**	**Agree**	**Disagree**	**Strongly disagree**	**Not sure**	
Adult intensive care unit staff	8%	45%	27%	4%	16%	**49**
Clinical Lead in organ donation	23%	66%	9%	0%	2%	**44**
Operating theatre staff	0%	36%	7%	7%	50%	**14**
Emergency care unit staff	0%	0%	0%	33%	67%	**6**
Other	15%	55%	5%	10%	15%	**20**
**Total**	**17**	**67**	**19**	**7**	**23**	**133**

#### NHS staff

There were concerns about variation in practice across the NHS and in specific areas (e.g. paediatrics and neonatal intensive care) and even discussing organ donation with some colleagues remained a challenge in these settings. CLODs we interviewed felt that the law had not helped and in some ways potentially created another hurdle, as the law did not apply to under 18s.

For many, the law change and its implementation were perceived as too abstract and subtle to figure highly on the NHS Trust agenda. The preference and focus for CLODs was to highlight more visible performance indicators especially related to organ retrieval and transplant. At a hospital level, this is what is used to try to increase the profile and priority given to organ donation but changing the law did nothing to help.

Intensivists confirmed that their main role(s) (and problems) were the steps before consent e.g. admitting and neurological death testing and that once this was working well on the intensive care unit, the pathway to organ donation is self-declared, which is unrelated to the law change and consent for organ donation, with consent remaining the SNODs/SRs job.

Some testimonies from intensivists cited poor previous experiences (e.g. a perception of withdrawal of treatment happening too soon or too late) as reasons why some colleagues did not (fully) engage with organ donation. Another factor for some intensivists was suddenly feeling left out of decision making, when there was a direct link created between NHSBT and the family at end of life, and observing the high(er) burden on families. The law in practice did nothing to reduce these issues and CLODs in particular reflected a tension between their role in promoting organ donation and maintaining or re-enforcing NHSBT standard operating procedures (SOPs) which are designed to keep organ donation separate from end-of-life care.

“*The thing that annoys me most is the doctor or nurse who accidently mentions ‘organ donation’ to the family. We know it’s not best practice and I can see the arguments, but I remember a time when no one was interested in organ donation, I used to pat everyone on the back in the group who mentioned it, but now you can’t do that. I do feel sorry for colleagues who feel that or accidently bring it up to the family, and then of course you have a big red flag over our unit, then I have to go and talk to them, and its like “I know you were working with the best intentions and I’m really grateful you’re thinking of organ donation but we think we get better consent rates if we do it this way and also these guys are the experts on organ donation. You only deal with them once a year, once every six months if you’re lucky”. Or I’d say unlucky because there is a lot of hassle involved in organ donation.” (clinician, interview)*

Other ITU staff were explicitly told that nothing had changed for them, not to worry and just keep doing what they had done before, as non-NHSBT staff did not have the same level of training to have these specialised conversations with families:

“*We actually have been told that we shouldn’t broach the conversation of organ donation to the family, it has to be through the SNOD. It probably is because we are not trained with the correct terms and how the conversation should go on” (ICU Nurse, interview)*.

Some felt the law posed a real threat to the work achieved (over 20 years) to clearly separate organ donation and end of life conversations, and it was this tension that many NHS staff reflected back, i.e. that the law was highly unlikely to have any positive impact on their day-to-day practice as they were not even able to discuss the prospect of organ donation before the law change, and so would not be discussing any change in consent policies after the law changed:

*“Referring [for potential organ donation] is straightforward but in the past I have found the organ donation nurses do not like it if we have spoken with the family regarding organ donation and several times have made me feel that I have overstepped the mark even after explaining that the subject was raised by the family.” (Second staff survey, Adult intensive care unit staff)*

In day-to-day practice, the lack of immediate access to a SNOD/SR and delays to their arrival on site amplified these tensions in terms of who does what and when since such delays were not always seen as in the best interests of the bereaved family:

“*In suitable cases [for potential organ donation] we sometimes have to wait a considerable time for the SNOD/[SR] (several hours) to arrive which can be very frustrating” (Second staff survey, Adult intensive care unit staff)*

#### NHSBT staff

SNODs/SRs self-identified as the key people for sustaining a culture of organ donation in the hospital. However, SNODs/SRs were also concerned about adding pressure or upsetting an overworked and burnt-out intensive care workforce. They particularly did not want to risk damaging relationships (built over time) by highlighting missed organ donation opportunities when cases were overlooked or not referred. A visible presence of SNODs was regarded as key (by everyone) in sustaining key performance indicators but this was frequently cited as a challenge due to staffing.

### Collective action: operational working together to achieve the common objective of implementing the new system

A large majority of respondents to the first staff survey (92%) agreed with the statement that they knew where to go to seek additional information and support material such as standard operating procedures and codes of practice on organ donation in their organisation. The first survey also revealed that over half (60%) of staff (not based in NHSBT) felt that NHSBT supported them in carrying out their tasks in relation to implementing the law change. This was highest among CLODs, where 98% of respondents responded that this was the case. Almost three-quarters of respondents to the first survey (73%) reported that they were completely or fairly confident in explaining the new law to patients and their family members. This, however, varied by group, with NHSBT staff showing higher levels of confidence than the other professional groups (see Table 8 for breakdown of responses by group). The more experience the SNODs/SRs had in deeming consent, the more the process became clearer to these staff and the more they supported the changes.

**Table 8:** Confidence in explaining the new law to patients and family members, by professional group (First survey)

**Professional group**	**Percentage within professional group**					**Total number**
	**Completely confident**	**Fairly confident**	**Somewhat confident**	**Slightly confident**	**Not confident at all**	
NHSBT staff	77%	20%	2%	1%	0%	**103**
Adult intensive care unit staff	4%	37%	22%	18%	18%	**49**
Clinical Lead in organ donation	40%	42%	9%	5%	5%	**43**
Operating theatre staff	0%	0%	44%	0%	56%	**9**
Emergency care unit staff	0%	17%	33%	0%	50%	**6**
Other	21%	42%	11%	21%	5%	**19**
**Total**	**102**	**66**	**25**	**16**	**20**	**229**

Just under half of the respondents to the second survey (49%) felt that they needed additional support, professional development or training to help them carry out their tasks in relation to implementing or sustaining the new system of organ donation in practice. This varied by professional group, with NHSBT staff being most likely to state they did not need such support or training. In terms of the type of additional support and training needed, the majority (58%) requested training (or refresher training) on how deemed consent was intended to work in practice, followed by training on issues affecting families from minority ethnic and different faith groups (42%). Additional requests included guidance on families’ decision-making powers as well as training on how to explain a diagnosis of death by neurological criteria to families. These concerns, about how the law was helping people work together were reflected in more detail in the interviews, below.

#### NHS staff

Many clinicians did reflect on how best practice was evolving during early implementation and the wider impacts NHSBT standard operating procedures might be having on realising the intended outcomes of the law change:

“I sometimes am worried that the push towards decoupling conversations with SNODs leads to disengagement [with organ donation] from clinicians. If you can go in a room and go yes, you’re going to die, over to you. But you never stay and find out what over to you is, and how you can help and influence the next stage. The whole point of it is to get the experts in a room together and work as a team, and it’s a very complex piece of team working because there is so much at stake. There’s obviously doing the best thing for the patient, that’s at stake. There’s a lot of pride on both sides and it is not very helpful at times. I think decoupling doesn’t always help because you create a very linear, my bit, your bit.. it is not really about the patient then is it. That’s just trying not to p*** each other off. It is also an impossible thing to accurately capture. because NHSBTs view is reliant on one individual’s presentation of how an interaction occurred, which is the SNOD/SR. And as we all know people interpret situations very differently from their different perspectives.” (CLOD, interview)

Similar inter-professional tensions were reflected by the nursing staff (particularly in London) earlier in the pathway:

“*We ‘suggest’,[referrals] we are always told that we can make a call ourselves, however, it would be quite difficult to work in that team when you bypass the clinical lead and, kind of, they feel like you’re going behind their back to make that call. Even though sometimes you actually see this person can save so many lives”. (ITU nurse, interview)*

#### NHSBT staff

SNODs/SRs continued prefer a highly personal and adaptable approach to potential donor families, but there were frustrations that the law had had little impact on typical issues they encountered on a daily basis. These are illustrated in Table 9.

**Table 9:** Typical issues the SNOD/SRs encountered on a daily basis identified in staff interviews

**Increased frustrations due to confused and mixed messages in the law**
*“As much as your trying to tell them you don’t need to make a decision, we are still asking families to support It [organ donation]. One minute we are saying, ‘deemed’ but then we can’t deem unless they support it [organ donation]. You are trying to say to them they’ve not opted-out so we want to deem consent, they’re objecting it, you push and push, and the family say, “Well if I don’t have a say what do I do?” And you’re having to say, “No, actually you do have a say, and if you say no, then that’s that…” (SNOD/SR)*
**Demotivation due to the law not elevating the importance of organ donation**
*“When I come out of that room and I can’t get consent, nobody cares, it’s so frustrating, the reality is I’m the only one out here searching for organs, if I don’t get them people die, its that simple really, I wanted it [law change] to help, it hasn’t” (SNOD/SR)*
**Deemed consent manifesting as nothing more than a tick box exercise**
*“A lot of families will come on board and go, ‘let’s go for it’, the deemed bit is only when I come to sign the form, so I’ll say something like, ‘the reason I’m signing this box is because your relative meets the criteria because they didn’t opt-out. But I’m still going to ask you to sign to say you’re supporting this’” (SNOD/SR)*
**Explaining not opting-out is choice which now means you have no objection to becoming an organ donor**
*“I can remember in a few conversations, families were saying to me, “He hasn’t done that intentionally. He did not know about the law change, so although his decision is blank, we have not discussed it, he does not know anything about it – he just sits in his chair every day and reads his book. We don’t even put the TV on. I’m telling you he has not actively left himself as a deemed. He just is what he was before, which is not on the Organ Donor Register.” How can you argue, we are not there to argue are we.”’ (SNOD/SR)*
**Potentially increased strain on professional relationships**
*“It’s tough we are trying to tell hospitals it’s [organ donation] normal but also ‘donation’, don’t talk about it. When I’m talking don’t speak, so is that the reason they are not backing you up, but then they [clinicians] come in with their own opinions and own level of comfortableness with deemed, and I think its hard, especially now when we don’t have the staff to man the units.” (SR/SNOD)*
*“My colleague who was with me [with the family], was like “I don’t know if you are aware [of the opt-out law now for organ donation] and she started explaining… Then one of the family reworded it, and said “she’s saying it is against the law if you don’t want to be a donor”, the family blew up, went mad. I wasn’t shocked [they were smoking, it stank of drink, I knew I needed to tread so carefully]. I would never have mentioned the law in front of this family because it looked like they wouldn’t have respected that, and they didn’t, they refused to speak to us again, it is hard families are so different aren’t they”. (SR/SNOD)*
*“We do have to mould them [clinicians] a little bit, some of them are a little bit green. It’s [mentioning organ donation] never done to wind us up or to push our buttons or anything like that. It’s done where they’ve thought they had a good inroad as part of the conversation when, actually, they should have just stopped that conversation there and allowed the family to digest it. Or the classic [the family say] “what happens next”, so again the over thinkers [clinicians] go, oh my god, I’m going to have to be honest and say it…organ donation…then we need to wind everything back” (SNOD/SR)*
**Disillusionment due to the lack of impact on highly individual and emotive situations**
*“We are dealing with irrational people, they are in crisis and grieving, trying to make a decision at that time is so hard, a sound decision, trying to apply the law at times of emotions, I would never ply it as a legal thing, I’d never leave them to believe they had no choice, or that it was happening irrespective of their suffering. The most authentic thing you can offer – is my experience, its comfort, giving hope and in time its [organ donation] meant something to so many, it’s about opening up that conversation and see how we do.” (SNOD/SR)*
**Irrelevant nuances in practice**
*“It feels a bit ridiculous, because Wales have a deemed law and we have a deemed law so the fact that you die on the wrong side of the border means you don’t apply the law [due to the residency status not allowing deemed consent to apply if people die outside their country of residence] if you’d been transferred to another hospital, it’s a bit frustrating, it seems stupid.” (SNOD/SR)*
**Concerns about (increasing) mistrust in the health system**
*“That’s the other dynamic we’re getting at the moment, since Archie Battersbee. We’re seeing a lot more resistance from families over withdrawal of treatment. The conversations that we’re having are so intense, lengthy conversations, far more questioning from families over decision making, treatment, length of treatment. Families are picking it apart, “How do you know he’s not going to get better? I’ve Googled it and this should be treated for 12 days!” (SNOD/SR)*
**The lack of clout in the law in traversing unyielding families**
*“The ones who are for it [organ donation] you’re just paying lip service to it [the law] by saying, thank you very much, obviously, you might be aware that the legislation supports you in this decision. And they’re just going, yes, get on with it, we want it, why are you telling me this, that’s lovely, bring out the forms. And the ones who are absolutely not going to entertain it [organ donation] are the types of families that it doesn’t matter what you say they will have an answer for everything. Oh you’re concerned about the operation, tell me about that. Actually, they wouldn’t want to do it for this reason, oh tell me, well actually, we’re not bothered, we’re not doing it!” (SNOD/SR)*
**Reconciling the law with acutely bereaved families**
*“I think law is a scary word for people and I know some colleagues of mine have used strong language when it comes to the law. Using the word law to people suggests there is going to be some sort of consequence should you not do it, so it becomes almost a threat. And on balance at a time of somebody’s acute grief that’s quite strong I think.” (SNOD/SR)*

#### NHS and NHSBT staff

On the whole, there was consensus that getting the donation conversation with the family right for everybody was a matter of the right staff coming together in the right ways and that this is something that is not easy to regulate, replicate or even articulate since the conversation has the capacity to shift and change course without warning with results which often remain uncertain and highly variable.

The general sentiment expressed was that this depended on the culture of the unit (often reliant on the embedded SNOD and a senior and enthusiastic CLOD), who else is on duty on the day and the limitations imposed by a permanently overstretched and overwhelmed workforce resulting in missed donation opportunities and lapses in best practice. Most continued to feel that until the public were more informed, their jobs would be no easier. Generally, work related to organ donation was perceived as above and beyond the normal standard of care:

“*You see with organ donation you have to have that drive and go over and above. We are asking people [NHS staff] to go over and above what they do, you’re asking favours, that’s how it feels a lot of the time and people are very nice about it and very kind but they’re obviously very, very busy with other patients. Then you get staff who say, this patient has died they’re not my priority, but I still need them [clinicians] to prescribe this, that and the other. So I do think from the hospital engagement side it’s just getting those powerful people who have that bit of a passion. From me what works really well, I’ve seen that there is a passion and there is a real interest and then that interest is fuelled in those [board] meetings and they just get really creative and they’re a strong force, they take it up and up and that’s what works best.” (SNOD/SR Interview)*

Reflexive monitoring: appraising the impact of the law and system changes on NHSBT and NHS staff, and the system

**Fig. 3:**
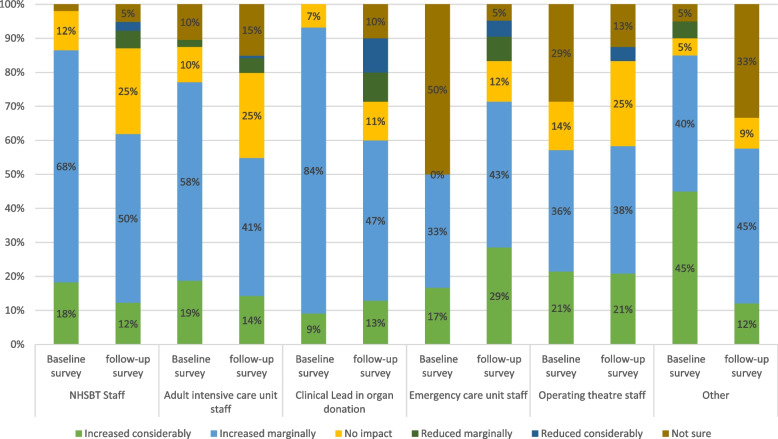
Perceived impact of initiatives and changes on consent rate for organ donation (first and second surveys)

**Fig. 4:**
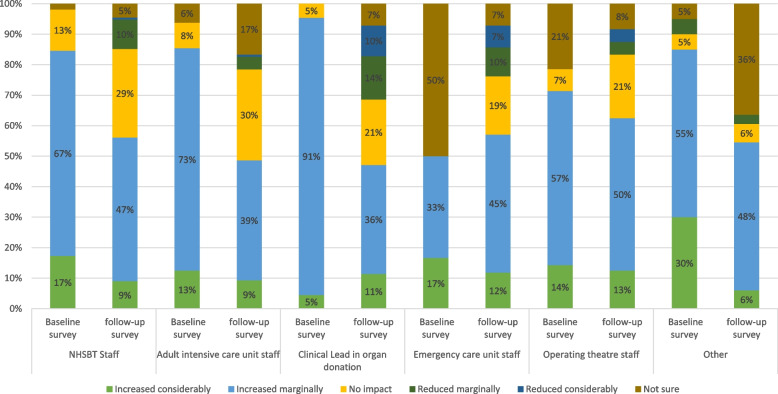
Perceived impact of initiatives and changes on number of organ donations (first and second surveys)

NHS and NHSBT staff receive regular feedback in terms of routine donor audit data. These audit data showed that the law change was not having the desired impact on organ donation consent rates in the initial implementation period. When comparing the two surveys, there was a corresponding decline in the percentage of respondents in the survey who perceived that the changes would result in an increase in consent rates, and an increase in those who believed the changes would reduce consent rates (see Figure 3). A similar trend was observed in relation to the perceived impact on the number of donations (see Figure 4). Support for the law in general also decreased over time (see Figure 2). When we asked respondents to indicate the main perceived benefits of the changes, the promotion of family discussions about organ donation was the most frequently selected (selected by 58% of respondents), followed by the perceived facilitation of organ donation discussions among staff (selected by 46% of respondents). Perceived downsides to the changes included that it made conversations difficult if relatives were not aware of the change in law. Almost half (49%) also selected the option that the law was too ‘soft’:

“*I don’t think it [law change] has had the desired impact on consent rates. In the deemed [consent] overrides that I have been [involved] in the law has been inconsequential to the family, as it is a soft law and cannot be enforced. The law needs to be hard and re-educated to the general public if it is to make a difference.” (Second staff survey, NHSBT staff)*

From a list of the top ten reasons identified in the 2021 NHSBT Annual Report on the Potential Donor Audit [22], the top three reasons why staff felt families still declined donation were that families felt that the length of time for the donation process was too long (46% of respondents); families were divided over the decision (45%); and the patient had previously expressed a wish not to donate (42%). To address these issues, staff most frequently advocated a media campaign to raise awareness among the public, as well as streamlining or shortening the processes involved in organ donation:

“*The amount of time it takes for SNODs to process potential organ donations is unacceptable and a reflection of serious under-staffing and increasing demands on their vetting procedures / work up required [screening and checking for organ donation potential]” (Second staff survey, Adult intensive care unit staff)*

**Fig. 5:**
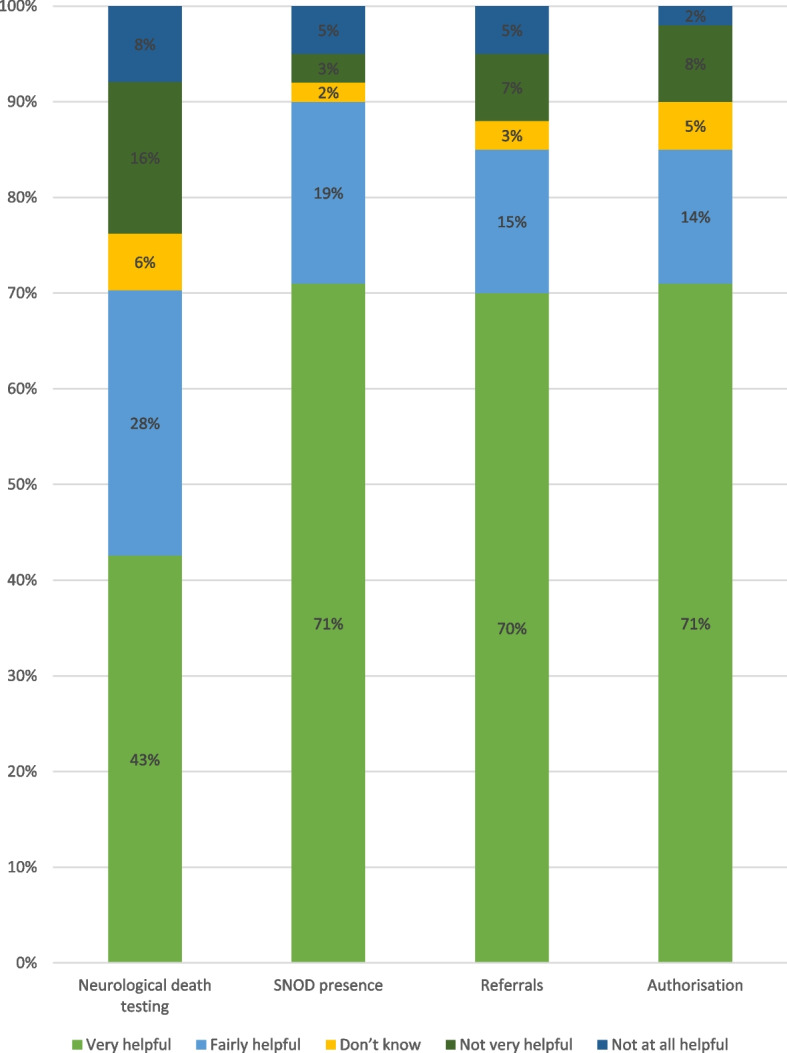
Views on NHSBT key performance indicators (second survey)

The majority of respondents indicated that all of NHSBT’s routinely collected performance indicators were helpful (see Figure 5 for details). In response to a follow-up question in the same survey, asking how these indicators could be changed, the highest number of responses received was in relation to neurological death testing, where respondents felt staff should not be penalised for not performing these tests when there was a valid reason not to do so (e.g. the patient was unstable). These decreasing trends in support for the law change and increase in frustration with the system were also prevalent in the interviews, as described below.

#### NHS staff

Some staff reflected a dilemma with trying to encourage brain death testing independently from organ donation as the two are so intertwined in practice and there were frequently frustrations on how death testing is captured in the audit data, its accuracy and relevance to missed organ donation potential.

There was a move towards organ utilisation as a priority area during the course of the study (due to the recent publication of recommendations on ways to maximise organ transplant from living and deceased donors). There was a consensus that poor utilisation dissuades wider NHS staff from engaging with organ donation (especially in hospitals) and that the law would do nothing to help this. Others reflected that devolved implementation is already causing problems in what are always evolving end of life practices and establishing national consensus and keeping guidelines up to date.

Staff in general felt that in order for anything to change, the impetus needed to come from a change in the public who would come to expect organ donation as a part of end of life. Many also reflected on the possibility that the law change was out of step with some sub-cultures in society as well as wider societal expectations and views on deceased organ donation, “If you want to do something in 10 years, interventions might (if you are lucky) get you there in 8-9 years, we basically asking people to be less religious, less sectarian less everything and this takes time.” (clinician interview)

Others discussed the quality of Organ Donor Register decisions, judging the extent to which decisions were adequately informed and proxy decision making, and that judging the quality of those decisions remained challenging. The decisions recorded on the register and the views of the family were viewed as integral to withdrawal of treatment and best interest conversations – nonetheless switching the law had done nothing to help these complex discussions on the pathway to organ donation. Some felt that in some ways NHS guidelines and NHSBTs standard operating procedures had gone too far by involving families too much in end of life decision making, and there was a genuine unease around declaring death using neurological criteria, related to some recent media cases around withdrawing treatment and declaring brain death [5, 23]. Some interviewees particularly in the Northwest on reflection felt that despite unpromising early evidence were reserving judgement because they thought it was too soon to reach a clear verdict on what difference the law change was making. Many in the Northwest were also concerned that the law was losing clout every time a potential organ donor case was overridden by the family and reflected that an order of priority should be:

“*We need to stop organ donor [register] overrides first, period. They [the deceased person] are on the [organ donor] register, there they are, not your [the family] decision, it’s happening. Then we can look into [verbally] expressed decisions and what they are or what not. We [SNODS and SRs] can’t do much about them at the moment, but it’s the deemed [consent] that’s the trouble. We are at least 20 years away from deemed[consent] being understood as a decision” (Clinician, interview)*

Most continued to feel that if there was no clear decision, then people would still take a cautious approach to organ donation irrespective of the change in law. Others highlighted that the law was never going to be good enough for such a diverse population:

“*We have the low hanging fruit as it were, the remaining 20-25% were always going to be a challenge, will the law [change] help, no, and we should accept that some families will continue to say no, no matter what we do” (Clinician, interview)*

#### NHSBT staff

SNODs/SRs felt the law change had quickly faded into the background and some were increasingly worried about the public’s knowledge of the changes and the messages to the public:

“*the message is now that you [the potential organ donor] do not have to do anything [to donate your organs] but families still don’t know [about deemed consent] and that really worries me, it feels like we are so far behind from Wales”. (SNOD, Interview)*.

Those who had been working in organ donation for a longer time (especially in the Northwest) were, however, disappointed at the lack of impact across the system and at an individual level:

“*it [law change] doesn’t give anymore reassurance to approaching [family members], we were nervous twenty years ago and we are nervous now. We wanted it to be a stronger more forceful, direct vehicle for change, but that hasn’t happened, I suppose in reality it all just takes time”. (NHSBT, interview)*

Most could see little change and felt, “that grief-stricken families can only take in so much, it is completely pointless to even try to explain the variables in consent, I mean ideally we want to be taking pressure off the families at really difficult times”. (SNOD/SR interview)

SNODs/SRs continuously reflected that the issues they were encountering were the same as before the law change and they continued to use their own individual interpersonal skills to “*schmooze and work with and around the various personalities*” they encountered. (SNOD Interview) It also remained very important to be seen as a supportive and helpful presence on the intensive care units:

“*At the start of my shift I’m supposed to go down and ask if there is anybody thinking about withdrawal of treatment. In the years I have been there is absolutely no way I would do that, I go down ask how everybody is, is there anything I can help with, would they like a cup of tea etc. then I can see what is going on and get invited into the discussions and get invited back, and that’s what changes things!”. (SNOD/SR interview)*

Everybody we interviewed said that the system issues were the same if not worse than before. Theatre space, funding, staffing, resources, training, reminding staff etc. remained untouched and were perceived to be at least if not more important than changing the law.

### Impact of COVID-19

The COVID-19 pandemic impacted every aspect of implementation and the organ donation service as a whole. The media campaign and formal launch were cancelled, all staff education and training was paused, all SNODs/SRs were redeployed to COVID-19 related activities and transplant services were severely disrupted. Unsurprisingly, our first survey (conducted between 08/2021 and 01/2022), indicated that over three-quarters of respondents (77%) said the pandemic had affected their ability to perform organ-donation-related tasks, to a great extent, or to some extent. A similar proportion (75%) indicated that the pandemic had affected their ability to perform their wider role within their organisation.

“*The roll out to staff not directly involved in organ donation was hindered by the pandemic, which dominated everything in ICU. No engagement with what SNODs could provide at the time in terms of training during the first COVID wave at it's peak - which was sometimes held virtually and was poorly attended. It led to a largely misinformed workforce - heard a lot of "everyone’s a donor automatically now" (First staff survey, NHSBT staff)*

In our second survey (conducted between 11/2022 and 01/2023), although most suggested that end of life care had returned to the pre-pandemic state, there were continuing disruptions, such as staff burn-out (including PTSD) (reported to be an issue by 26 respondents), reduced opportunities to interact with families, as a result of the restrictions that were still in place (reported by 25 respondents). An equal number, however, also identified positive changes, in the form of innovative adaptations to help with implementation (for example, videocalls with family members) that were facilitated as a consequence of the pandemic. Interview data reflected similar sentiments over time.

“*I do think we're coming out the other side [of the pandemic]. I do think the nation's returning to some kind of normal, but I think the hospitals and the staff are still terribly broken. And it feels like it’s just something that's going to just explode, if I’m honest with you. The staff are broken, so everyone else has moved on but then there’s no recognition for the people who worked right the way through it. It's now like, “Well you're not meeting these targets, you're not doing this, you're not doing that”. (SNOD/SR, interview)*

## Discusssion

### Principal findings

Our surveys of, and interviews with healthcare staff in England suggest general support for deemed consent of organs, but not for the manner in which it was implemented. They also identified a number of wider health-system issues that were perceived as barriers to the successful implementation of the change.

Although most staff felt prepared for the law change, losing SNODs’ embedded time within NHS hospitals was considered detrimental to relational work prior to, but exasperated by COVID-19. There were mixed messages and views about when SNODs/SRs should or should not be mentioning the ‘soft’ opt-out law change during their conversations with family members. SNODs/SRs shared that they often had negative experiences with families when talking through the law change and what it meant, which significantly affected their perceptions of the practicability of the law change. NHSBT staff sometimes found it challenging to make sense of, and distinguish, old from new practice, especially as the ‘soft’ opt-out was implemented into the existing opt-in system, neither system had universal coverage. After receiving initial training and education concerning the law change, many staff in the NHS and NHSBT were redeployed to help treat severely ill patients with COVID-19. This meant that there was an overall loss of opportunities for collective cognitive participation in implementing the law change because organ donation was not the priority during the pandemic. Overall, the Northwest region seemed to find it more straight forward to implement the change in law, in part because the SNODs/SRs in this region covered North Wales and had been working with the Welsh ‘soft’ opt-out system since 2015 and there were very active organ donation committees supporting implementation.

Many felt that the continued requirement from NHSBT imposed on other NHS staff not to mention organ donation to family members was harming collective action and caused frustration when staff felt punished for doing so, especially when they were trying to facilitate organ donation. Despite this frustration amongst NHS staff, SNODs’/SRs’ confidence with implementing the deemed consent pathway increased with the number of deemed approaches they had made.

There are many ongoing opportunities for reflexive monitoring in the organ donation system as NHSBT routinely collects a mass of data, which is fed back to all those involved in organ donation [24]. On the one hand, NHSBT appeared to be reassured that there was little difference in practice and consent rates following implementation of the law, due to anxieties that the law change and implementation during COVID-19 would make things worse, and on the other disillusioned that nothing had changed in their practice or their consent rates. They faced the same challenges as before – the law gave them no new tools to navigate the complexities of speaking to the acutely bereaved or influencing the family’s behaviours in regard to deceased organ donation. NHS clinicians too felt that NHSBTs standard operating procedures did not easily reflect reality on the ground, and were not always helpful in what were unique and complex, discussions and negotiations.

### Meaning of this study

Our study has shown the complexities of trying to bring about change in a system where the key implementers (SNODs/SRs) are sometimes only in post for a short-term, have less time and resources than in the recent past to promote an activity (organ donation) which, for the majority of NHS staff, is very rare. This means that it is difficult to achieve coherence, cognitive participation and collective action to support implementation that is meaningful and sustainable across the NHS and NHSBT. This was made especially challenging to achieve in the unique context of the COVID-19 pandemic.

Over time, support for the law change decreased as did any perceived positive impact the law might have on consent or the organ donation system. In their reflexive monitoring and appraisals, staff continued to feel that the reasons for refusals were the same (processes too long, family divided or had previously said they did not want to donate). The lack of clout of the law, its limited capacity to cope with population heterogeneity, irrelevant nuances in the law in practice, and the lack of impact on end-of-life proxy decision-making gave no more reassurance to anyone that the law would work in a practical help anybody in the system. Organ donation remains relatively rare even for ICU staff and sits outside clinical care of patients and thus requires staff involved to go to great efforts to secure donations, and within a permanently overstretched system and overworked workforce, making organ donation a priority outside NHSBT continued to prove challenging.

Introducing an opt-out policy in England automatically switched the default position of nearly 45 million adults to one that, in principle, should have positively supported organ donation. However, in practice, this has resulted in a series of standard operating procedures trying to cover a whole range of processes and scenarios and are now standing in direct opposition to the aims of the changes, which were to make organ donation a routine part of end-of-life care. While staff on the frontline remained highly motivated and engaged with organ donation and the good it might bring about, the lack of evidence of a positive effect on consent rates contributed to staff becoming disillusioned with the law change and any potential good it might bring about.

### Implications for policy and practice

The findings presented in this paper are consistent with similar research in other countries which indicates deemed consent has had positive impacts on consent rates in some countries and negative impacts in others [5, 25], and that knowledge and awareness of healthcare staff about deemed consent and how it is intended to work is generally low [26] Due to the mixed evidence, it is too soon to tell whether England is likely to be one of the positive cases but without the additional implementation strategies (discussed above) impact (on consent rates) will likely remain marginal.

It seems appropriate now that the crisis phase of the pandemic is over to take stock and consider what would further enhance implementation of the law change. When thinking about further changes and enhancements that could be made to the current ‘soft’ opt-out system three years after initial implementation, findings suggest that it would be helpful to revive the programme of support for the law change, which was cancelled due to the pandemic, with a focus on rebuilding and stabilising the NHS and NHSBT workforce involved in organ donation in the wake of COVID-19. Other initiatives to consider include revising and relaunching training and renewing the public media campaign. It will likely be challenging to move organ donation up the NHS priority list when there is an ongoing staffing crisis, staff are striking for more pay and there is a huge backlog of patients requiring treatment, but it is clear that unless organ donation becomes a higher priority, it will be difficult to bring about the required changes. NHS staff outside NHSBT, in particular, need to become more involved in deceased organ donation, to be encouraged to believe that it is their business to be involved in organ donation and to be provided with more stimulating work processes (e.g. examining the NHSBT veto on clinical staff becoming more involved in organ donation conversations) in order to stay motivated to maximise organ donation opportunities. Operational processes need to adapt to the intentions of the new legislation and ‘soft’ opt-out, rather than compete with it. Markers of success need to be individually tailored and more meaningful (e.g. number of lives saved or improved) and not just on establishing what is and is not deemed consent. Overall, current processes and operating procedures need to be changed such that they make implementation of the ‘soft’ opt-out easier and create simpler work processes for everyone.

### Strengths and limitations

This is the largest real-time evaluation of the implementation of a switch from opt-in to opt out organ donation legislation. The highly novel and rigorously conducted study was timed to coincide with the initial implementation phase so that memory recall was recent. It involved large numbers of purposively selected staff interviews and survey responses, which when integrated provide a detailed picture of the implementation challenges. Findings will have an impact as they provide the first robust evidence on the ineffective initial implementation phase and a clear steer on how to optimise implementation and further increase consent rates.

Some of our data collection was hampered by COVID-19, in particular the low response to the first survey, but we recruited a high percentage (approximately 52%) of relevant NHSBT staff (SRs, SNODs and managers) and CLODs (approximately 70%) to the second survey. It was not possible for us to calculate a response rate to the surveys for reasons described above, which also meant we could not establish how representative our sample was of the population whose views we were seeking. Another limitation of our research was that some follow-up interviews were paused as there was very little evidence of impact or change over time. NPT was helpful in visualising and integrating data and explaining why the ambitions of the Act were not yet coming about in practice.

## Conclusion

Implementing the law change at the height of the pandemic and in a crisis situation when many staff were retrained and redeployed elsewhere has meant that implementation strategies were ineffective, diluted or did not happen. Although broadly supportive of the law changes as morally the right thing to do, NHSBT staff were not generally convinced that the ‘soft’ opt-out system would deliver the expected increased consent rates as envisaged by legislators. NHS staff, in particular, were not able to fully consider or process the required changes to implement the ‘soft’ opt-out legislation during the pandemic due to other competing priorities and general disruption to care as usual. The NHS now needs to reprioritise organ donation (although this will be challenging given the current pressures on the NHS) and relaunch and revise the continued implementation of the ‘soft’ opt-out system with a largely (albeit slowly) replenished workforce. Nonetheless, even with a relaunched implementation a ‘soft’ opt-out system is always going to be difficult to implement if the main goal is to significantly increase consent rates.

## Supplementary Information

Supplementary Material 1.

## Data Availability

The datasets used and/or analysed during the current study available from the corresponding author on reasonable request.
