# Supplementary material for: Perceptions and experiences of healthcare professionals of implementing the Organ Donation (Deemed Consent) Act in England during the Covid-19 pandemic
Source: BMC Health Serv Res. 2025 Jan 31;25:183. doi: 10.1186/s12913-025-12224-8 (PMC11786446; doi:10.1186/s12913-025-12224-8)
Supplement: Supplementary file 1 — Supplementary Material 1. [file 12913_2025_12224_MOESM1_ESM.docx]

**Appendix 1:**

**Organ Donation policy package**

**Organ Donation (Deemed Consent Act) 2019**

Change in the law introducing the concept of “deemed consent” from spring 2020 – in the absence of a decision to donate or not donate, the presumption will be in favour of organ donation.

**National communication campaign about law change**

In April 2019, NHSBT started the national awareness campaign about the changes in the law from 2020. This has focused on using social media, radio adverts, information available on NHSBT’s website etc and will ramp up in the new year with TV adverts ahead of May 2020 (our go live date is 26 May 2020 but it is not in the public domain).

**Targeted communication campaign about law change**

Alongside the national awareness campaign, NHSBT is also targeting specific groups such as BAME, faith groups and those approaching 18.

**NHS Organ Donor Register (ODR)**

NHS Database where someone records a decision about organ donation (to opt-in or opt-out). A key message of the communication campaign is to record a decision as research shows that when families know what their relative would have wanted they are more likely to support their decision.

**Specialist Nurses for Organ Donation/Specialist Requesters/Specialist Nurses-Tissue Donation (SNTD) - training**

Specialist Nurses are trained to discuss organ donation with families if organ donation is a possibility for their relative and support them throughout the process by answering questions etc. The Specialist Nurses will have a key role in the opt-out.

**NHS App**

New NHS App just launched making it possible to also access the ODR through it.

**Government public consultations**

Government consulted on two occasions on the move to an opt-in system – the first consultation received 17,000 responses and the second one 4000. Many stakeholders took a key interest in the new policy, including representatives of faith groups

**Code of Practice for healthcare professionals**

Practical guidance to healthcare professionals. There will be updated guidance for healthcare professionals about how the system will work in practice. Issues to be covered will be how to establish if someone has made a decision, what is the role of the family, how to establish if someone is excluded from deemed consent etc.

**Amending clinical protocols and procedures**

NHSBT have a number of protocols and procedures in place about the donation process.

Procedures and protocols have been updated to reflect the new system

**Training of NHSBT healthcare staff on the new system**

NHSBT healthcare professionals are trained on issues around consent etc. This includes the Specialist Nurses, retrieval teams etc. Updated training to cover the new system. This will include faith training and development of resources in the form of a DAT, information leaflets and YouTube links. This ties in with person centred care and adopting a humble approach to end of life and/or care after death practices.

**Introduction of Specialist Requestors**

New role for Specialist Requestors for organ donation, focussing on liaising with/ supporting the family rather than clinical activity. This was an existing programme to roll out to all teams after a successful pilot.

**Changes to the Potential Donor Audit**

Amendments to the Potential Donor Audit to collate data from Trusts about people dying in intensive care. The type and process for data collection is changing to support new initiatives.

**National Call Centre capacity and training**

To ensure that they can keep pace with increases in calls to the ODR call line, so that queries and registration requests are managed quickly and efficiently; and that the call centre team are aware.

**Appendix 2: Staff surveys**

**First survey**

Section 1: **General information about respondent**

Q1: Which of the following professional groups do you belong to, in relation to organ donation?

- NHSBT staff
- Clinical Lead in organ donation
- Adult intensive care unit staff
- Emergency care unit staff
- Operating theatre staff
- Other: Please specify

Q2: Which NHS trust do you work for in relation to organ donation? Your answer to this question will enable us to report the percentage of eligible NHS Trusts from which we received a response, and will not be used when we report the findings. For Specialist Nurses in Organ Donation, please indicate the trust where you hold an honorary contract.

[List of Acute (Hospital) Trusts in England](https://www.nhs.uk/servicedirectories/pages/nhstrustlisting.aspx)

Q3: How long have you been in your current post (please answer in relation to organ donation)?

- Less than a year
- Between 1-3 years
- More than 3 years

Section 2: **Awareness and understanding of the law change**

Q4: How well do you feel that you understand the recent law change in organ donation in England?

- I am clear in my understanding about the new change in the law in organ donation.
- I am mostly clear in my understanding about the new change in the law in organ donation, but am unsure on some aspects.
- I am not clear about the organ donation law change.

Q5: The law change in organ donation was supported through other initiatives and health system changes. Please indicate those which you are aware of from the list below:

| Changes/ initiatives | Aware of | Not aware of | Not sure | Not applicable |
| --- | --- | --- | --- | --- |
| General public media campaign (social media, TV & radio adverts, health Websites) |  |  |  |  |
| Communication campaign targeted at groups (e.g. ethnic minorities, young people, Faith groups) |  |  |  |  |
| Expansion of the workforce of Specialist Practitioners (Specialist Requesters in organ donation) who are responsible for obtaining consent to organ donation from family members of the deceased person. |  |  |  |  |
| Introduction of app feature to register organ donation decision through the NHS App |  |  |  |  |
| Updated Human Tissue Authority Code of Practice for Organ Donation for healthcare professionals |  |  |  |  |
| Amended NHSBT clinical protocols and procedures for organ donation |  |  |  |  |
| Training of NHSBT healthcare staff on new system |  |  |  |  |
| Training of other NHS staff (e.g. emergency department, critical care) on referrals for organ donation |  |  |  |  |
| Changes to the ways data are collected and shared between NHS and NHSBT about people who are potential organ donors, e.g. the Potential Donor Audit |  |  |  |  |
| Information and guidance on organ donation from professional bodies to healthcare professionals |  |  |  |  |
| Enhancing national call centre capacity and training to keep pace with likely increase of calls to the Organ Donation Register line’. Please find further details of these initiative [here](https://lshtm.sharepoint.com/:w:/r/sites/OrganDonation/Shared%20Documents/scoping%20study/Organ%20Donation%20policy%20package.docx?d=w7707b6297fde44969f00c55f97458094&csf=1&web=1&e=pjxPo5) |  |  |  |  |

Q6: (Carry over responses selected from Q5) Did you find the following initiatives and health system changes helpful in your understanding of the guidelines on organ donation?

| Change/ initiatives | Helpful in my understanding | Not helpful in my understanding | Not sure | Not applicable |
| --- | --- | --- | --- | --- |
| General public media campaign (social media, TV & radio adverts, health Websites) |  |  |  |  |
| Communication campaign targeted at groups (e.g. ethnic minorities, Young people, Faith groups) |  |  |  |  |
| Introduction of Specialist Practitioners (Specialist Requesters in organ donation) who are responsible for obtaining consent to organ donation from family members of the deceased person. |  |  |  |  |
| Introduction of app feature to register organ donation decision through the NHS App |  |  |  |  |
| Updated Human Tissue Authority Code of Practice for Organ Donation for healthcare professionals |  |  |  |  |
| Amended NHSBT clinical protocols and procedures for organ donation |  |  |  |  |
| Training of NHSBT healthcare staff on new system |  |  |  |  |
| Training of other NHS staff (e.g. emergency department, critical care) on referrals for organ donation |  |  |  |  |
| Changes to the Potential Donor Audit |  |  |  |  |
| Information and guidance on organ donation from professional bodies |  |  |  |  |
| National Call Centre capacity and training |  |  |  |  |

Q7: (Carry over responses selected from Q5) What difference did the following initiatives and health system changes make to your practice regarding organ donation?

|  | Made my practice easier | Made no difference to my practice | Made my practice harder | Not sure | Not applicable |
| --- | --- | --- | --- | --- | --- |
| General public media campaign (social media, TV & radio adverts, health Websites) |  |  |  |  |  |
| Communication campaign targeted at groups (e.g. ethnic minorities, Young people, Faith groups) |  |  |  |  |  |
| Introduction of Specialist Practitioners (Specialist Requesters in organ donation) who are responsible for obtaining consent to organ donation from family members of the deceased person. |  |  |  |  |  |
| Introduction of app feature to register organ donation decision through the NHS App |  |  |  |  |  |
| Updated Human Tissue Authority Code of Practice for Organ Donation for healthcare professionals |  |  |  |  |  |
| Amended NHSBT clinical protocols and procedures for organ donation |  |  |  |  |  |
| Training of NHSBT healthcare staff on new system |  |  |  |  |  |
| Training of other NHS staff (e.g. emergency department, critical care) on referrals for organ donation |  |  |  |  |  |
| Changes to the Potential Donor Audit |  |  |  |  |  |
| Information and guidance on organ donation from professional bodies |  |  |  |  |  |
| National Call Centre capacity and training |  |  |  |  |  |

Q8: How confident are you in explaining the new law on organ donation to patients and their family members?

- Not confident at all
- Slightly confident
- Somewhat confident
- Fairly confident
- Completely confident
- Not applicable

Q9: How confident do you feel in your day-to-day application of the new law? (i.e. in that you are acting within the current law on organ donation)

- Not confident at all
- Slightly confident
- Somewhat confident
- Fairly confident
- Completely confident
- Not applicable

Q10: To what extent do you agree with the statement: ‘I know where to go to seek additional information and support material such as clinical protocols and codes of practice on organ donation in my organisation’?

- Strongly agree
- Agree
- Neither agree nor disagree
- Disagree
- Strongly disagree
- Not sure/ Don’t know

Q11: Please indicate below the source of any training/ professional development, or support you received on donation law before and after the law change, and the timing of training

| Source of training/ professional development or support | Timing of training/ professional development or support | |
| --- | --- | --- |
|  | Before the law change | After the law change |
| Employing organisation (in-house) |  |  |
| Professional body/ association |  |  |
| NHSBT (only asked of non-NHSBT staff) |  |  |
| Other? |  |  |

Q12: If indicated that they received any training in response to Q11: Has the training you received changed your practice in any way?

- Yes
- No
- Cannot remember/ Not sure

Q13: If answered “Yes” to Q12, then ask: Please describe in the box below how the training has changed your practice.

Free Text

Q14: To be asked of non-NHSBT staff: To what extent do you feel NHSBT has supported you in carrying out your work in relation to the law change on organ donation?

- To a great extent
- To some extent
- To a little extent
- Not at all
- Not sure

Q15: To be asked of NHSBT staff: To what extent do you feel you have received sufficient training, professional development and support in carrying out your tasks in relation to the law change on organ donation?

- To a great extent
- To some extent
- To a little extent
- Not at all
- Not sure

Q16: To be asked of non-NHSTBT staff: Please say whether you agree or disagree with the two statements below in relation to preparing for the law change on organ donation:

|  | Strongly agree | Agree | Disagree | Strongly disagree | Not sure |
| --- | --- | --- | --- | --- | --- |
| I feel that I was adequately prepared for the change in the law on organ donation |  |  |  |  |  |
| I feel that my organisation was adequately prepared for the change in the law on organ donation |  |  |  |  |  |

Q17: Please describe any additional support, professional development or training that would help you carry out your tasks in relation to organ donation:

Free text box

Section 3: **Degree of support for the new organ donation law and reason for supporting/ not supporting**

Q18: How supportive are you of the changes to the deemed consent law in England?

1. Not supportive at all
2. Somewhat unsupportive
3. Neither supportive nor unsupportive
4. Somewhat supportive
5. Completely supportive

Q19: If responded 1 to Q18: Why did you respond that you were “*not supportive at all”* of the changes to the deemed consent law in England?

Free text box

If responded 5 to Q18: Why did you respond that you were *completely supportive* of the changes to the deemed consent law in England?

Free text box

Q20: What impact, if any, do you think the changes to the organ donation law and health system will have on the following indicators:

|  | Increase considerably | Increase marginally | No impact | Reduce marginally | Reduce considerably | Not sure |
| --- | --- | --- | --- | --- | --- | --- |
| Consent rate |  |  |  |  |  |  |
| Number of donations |  |  |  |  |  |  |

Q21: Have you experienced any downsides to the changes to the organ donation law and the associated changes with the health system? Please describe any downsides in the box below:

Free text box

Section 4: **COVID-19**

Q22: To what extent has the COVID-19 pandemic affected the following aspects of your role:

| Aspects of role | To a great extent | To some extent | To a little extent | Not at all | Not sure | Not relevant |
| --- | --- | --- | --- | --- | --- | --- |
| ability to perform organ donation-related tasks |  |  |  |  |  |  |
| ability to perform wider role within organisation |  |  |  |  |  |  |

Section 5: **Other comments**

Q23: Please use the box below to describe any additional changes you think could improve organ donation rates in England:

Free text box

Section 6: **Follow up survey**

Q24: We are planning a follow-up survey in 12-18 months’ time. It would be very helpful for our research if we could capture your views then. Can we please contact you then?

- Yes
- No

Q25: If answered Yes to Question 24. Can you please provide us with an e-mail address that we can use to reach you for the follow-up survey? This will only be used for this purpose.

**Second survey**

Section 1: **General information about respondent**

Q1: Which of the following professional groups do you belong to, in relation to organ donation?

- NHSBT staff
- Clinical Lead in organ donation
- Adult intensive care unit staff – Nursing staff
- Adult intensive care unit staff – Medical staff
- Emergency care unit staff
- Operating theatre staff
- Other: Please specify

Q2: Which NHS trust do you work for in relation to organ donation? Your answer to this question will enable us to report the percentage of eligible NHS Trusts from which we received a response, and **will not be used when we report the findings or for any other purposes**. For Specialist Nurses in Organ Donation, or Specialist Requestors, please indicate the trust where you hold an honorary contract.

[List of Acute (Hospital) Trusts in England](https://www.nhs.uk/servicedirectories/pages/nhstrustlisting.aspx)

Q3: How long have you been involved in organ donation?

- Less than a year
- Between 1-3 years
- More than 3 years

Section 2: **Awareness, understanding and implementation of the law change**

Q4: How well do you feel that you understand the law change in organ donation in England, which came into effect in May 2020?

- I am clear in my understanding about the change in the law in organ donation.
- I am mostly clear in my understanding about the change in the law in organ donation, but am unsure on some aspects.
- I am not clear about the organ donation law change.

Q5: Do you require any additional support, professional development or training to help you carry out your tasks in relation to organ donation?

- Yes
- No
- Unsure

Q6: (if answered “Yes” to Q5 above, then ask): What additional support, professional development or training would help you carry out your tasks in relation to organ donation? Please select all that apply

- Training on how to communicate with family members/ next of kin
- Training/ refresher training on how deemed consent is intended to work in practice
- Mentoring on how to carry out tasks
- Clinical supervision when carrying out tasks
- Something else: Please describe in the box below

Free text box

Q7: Do you think the deemed consent legislation is now part of normal practice in your organisation?

- Yes
- No
- Unsure

Section 3: **Degree of support for the new organ donation law and reason for supporting/ not supporting**

Q8: How supportive are you of the change to the deemed consent law in England?

1. Not supportive at all
2. Somewhat unsupportive
3. Neither supportive nor unsupportive
4. Somewhat supportive
5. Completely supportive

Q9: Since the introduction of the change to the law on organ donation in England in May 2020, has your support for deemed consent…

- Increased
- Not changed
- Decreased
- Not sure

Q10: If responded ”increased” to Q9: Why has your support for deemed consent **increased**?

Free text box

Q11: If responded “decreased” to Q9: Why has your support for deemed consent **decreased**?

Free text box

Q12: The law change in organ donation was supported through initiatives and changes to the system of organ donation, such as the expansion of the workforce of Specialist Requestors who are responsible for obtaining consent to organ donation from family members of deceased persons (the full list can be accessed here). What impact, if any, do you think these initiatives and changes have had so far?

|  | Increase considerably | Increase marginally | No impact | Reduce marginally | Reduce considerably | Not sure |
| --- | --- | --- | --- | --- | --- | --- |
| Consent rate for organ donation |  |  |  |  |  |  |
| Number of donations |  |  |  |  |  |  |

Q13: Have you experienced any **benefits** to the change to the organ donation law and the associated changes with the health system, introduced in May 2020? Please select all that apply from the list below:

- It prompts family discussions
- It facilitates organ donation discussions among staff
- It empowers patients to make decisions about their organs
- It simplifies my role/ gives my role more clarity
- Something else: please use the box below to describe these upsides

Q14: Have you experienced any **downsides** to the change to the organ donation law and the associated changes with the health system, introduced in May 2020? Please select all that apply from the list below:

- The law adds another layer of bureaucracy
- Makes conversations difficult if relatives are not aware of change in law
- The law is too soft- relatives can override the patient’s decision
- Something else: please use the box below to describe these downsides

Section 4: **Improving Organ Donation rates in minority ethnic groups and faith groups**

Q15: Organ donation rates are lower in some minority ethnic groups and from certain faith perspectives. What impact, if any, did the changes have on gaining consent from family members from these groups?

1. It made gaining consent easier
2. It did not have any impact on gaining consent
3. It made gaining consent harder
4. Not sure

Q16: If answered “*It made gaining consent easier*” in response to Q15, then ask: Please describe below how the changes made gaining consent **easier**

Free text box

Q17: If answered “*it made gaining consent harder*” in response to Q15, then ask: Please describe below how the changes made gaining consent **harder**

Free text box

Section 5: **Non-consenting families**

Q18: The 2021 NHSBT Annual Report on the Potential Donor Audit identified the following top ten reasons families did not give consent for the donation of their deceased relative’s organs. Please select the three most common reasons you have encountered during conversations with families. If this includes reasons not on the list below, then please select “Other” and describe them in the box.

- Patient had previously expressed a wish not to donate
- Family felt it was against their religious/ cultural beliefs
- Family were not sure whether the patient would have agreed to donation
- Family did not want surgery to the body
- Family felt patient had suffered enough
- Family divided over the decision
- Family felt that the body should be buried whole (unrelated to religious/ cultural reasons)
- Family did not believe in donation
- Family felt that the length of time for the donation process was too long
- Patient had registered a decision to opt out
- Other: Please describe in the box.

Q19: What changes do you think can be introduced to the organ donations system to address these reasons?

Free text box

Section 6: **COVID-19**

Q20: What impact, if any, is the COVID-19 pandemic having on the routine part of End-of-Life care in your organisation, in the post-pandemic recovery stage?

Free text box

Section 7: **Targets**

Q21: NHSBT collects and reports data on a number of [key performance indicators](https://app.powerbi.com/view?r=eyJrIjoiMGIxYWIyMmEtZmU0Ny00OTkwLTlmNWUtYmEyNmFlZDI1YmQyIiwidCI6ImUxMWExYjhmLThmNTItNDYwOC04Zjc2LTQ2N2ZmMWQzNGM5NiIsImMiOjh9) of the activity of Trusts/ Boards across the UK. To what extent (if any) do you find each of these indicators are helpful in measuring performance in relation to organ donation?

| **KPI** | **Views** | | | | |
| --- | --- | --- | --- | --- | --- |
|  | **Not at all helpful** | **Not very helpful** | **Fairly helpful** | **Very helpful** | **Don’t know** |
| **Neurological death testing** |  |  |  |  |  |
| **SNOD presence** |  |  |  |  |  |
| **Referrals** |  |  |  |  |  |
| **Consent/ Authorisation** |  |  |  |  |  |

Q22: Would you like to see any changes to the way these key performance indicators are collected or used?

| **KPI** |  | | |
| --- | --- | --- | --- |
|  | **Yes** | **No** | **Not sure** |
| **Neurological death testing** |  |  |  |
| **SNOD presence** |  |  |  |
| **Referrals** |  |  |  |
| **Consent/ Authorisation** |  |  |  |

Q23: If answered “Yes” to any of the KPIs in last question, then ask: You indicated that you would like to see changes to how (name of KPI) is collected or used. Please use the box below to describe what these changes would be.

Free text box

Section 8:  **Other changes to the OD System**

Q24: Please use the box below to describe any changes that would make your role (in relation to identification, referral and consenting for organ donation) easier to perform?

Free text box

Q25: Please use the box below to describe any additional changes you think could improve organ donation rates in England:

Free text box

**Appendix 3: NPT analytical framework**

| **Construct/ subconstruct Name** | **Description** | **Example of evidence in Organ Donation evaluation** |
| --- | --- | --- |
| **COHERENCE**  ***Coherence*** *is the* ***sense-making work*** *that people do individually and collectively when they are faced with the problem of operationalizing some set of practices.* | **MAKING SENSE OF IT** The extent to which study participants had clear knowledge and understanding of the change in the law (the intervention). Sense making of new practices - the meaningful qualities of a practice. |  |
| Differentiation  *An important element of sense-making work is to understand how a set of practices and their objects are different from each other.* | Do people across the area see a coherent model and distinguish it from current ways of working? Do they see this as a new way of working? Or is this business as usual? How is what is being implemented different from what already happens? Are people able to see a difference between the old and new practices - can they differentiate between what happened before and now | What’s changed/different  *Capture all experiences, descriptions actions, activities that have changed/are different* (or not) as a result of opt-out from the multiple perspectives. |
| Communal specification Sense-making relies on people working together to build a shared understanding of the aims, objectives, and expected benefits of a set of practices. | Do people collectively agree about the purpose of the intervention? what does the intervention mean for team working? How will the new system change the current work of the team? Is there a collective or shared idea of the change in the law and the practice around that - e.g. between NHSBT and NHS Staff in different departments and areas of work - good integration of practice (are SRs and CLODS working with other clinicians to integrate this into practice) | Awareness/understanding/  support (team, wider service, systems) *Capture experiences, thoughts, descriptions, of the ways opt-out is working (or not) from a team wider/service perspective* |
| Individual specification *Sense-making has an individual component too. Here participants in coherence work need to do things that will help them understand their specific tasks and responsibilities around a set of practices.* | Do individuals understand what tasks the intervention requires of them? what does the change in the law mean for specific people (how do individuals understand how the OD affects their work). Do people on the whole understand the new consent law and how it is intended to operate. | Awareness/understanding/  Support (personal, individual job role) *Capture experiences, thoughts, descriptions, of the ways opt-out is working (or not) for individuals* |
| Internalisation  *Finally, sense-making involves people in work that is about understanding the value, benefits and importance of a set of practices* | Do all the stakeholders grasp the potential benefits and value of the intervention for their work? Do they support it? Are people attributing work to the change in the law and taking ownership - are they internalising the new practice - what processes are in place to stimulate this internalisation? e.g. communicating evidence of progress - data on consent rates) or informal discussions on progress and impact of the law change | What difference will it make (experiences, opinions, thoughts on what difference opt-out will make, on whom and why) |
| **COGNITIVE PARTICIPATION** | **WORKING OUT PARTICIPATION – ‘BUY IN’** The extent to which participants bought in to the law change, engaged with it, and committed to it (training modules). Establishing relationships and divisions of labour to support the intervention (enrolment and engagement of individuals and groups) – “What does good look like” |  |
| Initiation  *When a set of practices is new or modified, a core problem is whether or not key participants are working to drive them forward.* | WHO IS DRIVING THIS? Are they willing and able to engage others in the implementation? Who are the key people and what are they doing? How is the new practice initiated? Is there high-level formal agreements to implement and make resources available among leaders - clear arrangements (formal) steering groups, implementation plans and governance | Normalising organ donation (experiences views and actions of making organ donation a normal/routine part of end of life care) |
| Enrolment  *Participants may need to organize or reorganize themselves and others in order to collectively contribute to the work involved in new practices. This is complex work that may involve rethinking individual and group relationships between people and things.* | DO PEOPLE AGREE THIS SHOULD BE PART OF THEIR WORK? How are people enrolled forming and organising how participants join new practices - any new roles - any boundaries hindering enrolment. Do the stakeholders believe they are the correct people to drive forward the implementation? how do participants become involved in the intervention (how are staff made aware – trained) | Concerns/problems (what’s stopping organ donation becoming normal from multiple perspectives) |
| Legitimation  An important component of relational work around participation is the work of ensuring that other participants believe it is right for them to be involved, and that they can make a valid contribution to it | DO PEOPLE BUY IN TO IT? Do they believe it is appropriate for them to be involved in the intervention? why should a participant participate? How is legitimacy established? How staff work to shape their role and establish legitimacy | Influences/Influencers (experiences, activities and descriptions of influences on opt-out and associated processes e.g. training, transplant, live donation) |
| Activation  Once it is underway, participants need to collectively define the actions and procedures needed to sustain a practice and to stay involved. | DO PEOPLE CONTINUE TO SUPPORT THE INTERVENTION? Can stakeholders identify what tasks and activities are required to sustain the intervention? What processes will support people staying on task - making it work well. | Motivation to stay involved (experiences, views and activities of people’s motivations to change/adapt) |
| **COLLECTIVE ACTION**  Collective Action is the **operational work** that people do to enact a set of practices, whether these represent a new technology or complex healthcare intervention. | **DOING IT** The work done by individuals and organisations to enact the new practice. The allocation or organisational and personal resources to support the change in the law and how this has been operationalised - how roles and responsibilities are defined. The operational work of implementation (interaction with already existing practices. IMPLEMENTATON |  |
| Interactional workability, *This refers to the interactional work that people do with each other, with artefacts, and with other elements of a set of practices, when they seek to operationalize them in everyday settings.* | DO ALL PEOPLE INVOLVED CLEARLY PERFORM THE TASKS REQUIRED FOR THE INTERVENTION? Does the intervention make it easier or harder to complete tasks? how does the intervention affect existing working practices and relationships? (is it disruptive?) | Experiences of implementing opt out*(capture actual experiences of implementing opt-out, associated policies, procedures, practices)* |
| Relational integration, *This refers to the knowledge work that people do to build accountability and maintain confidence in a set of practices and in each other as they use them.* | DO PEOPLE TRUST EACH OTHERS WORK AND EXPERTISE IN THE INTERVENTION? Do those involved in the implementation have confidence in the new way of working? how are confidence in, and accountability for the intervention built? | How are people adapting  (*capture, experiences, views and activities of the ways people are adapting to optout)* |
| Skills set workability,  *This refers to the allocation work that underpins the division of labour that is built up around a set of practices as they are operationalized in the real world* | IS THERE APPROPRIATE ALLOCATION OF WORK? Do those implementing the intervention have the correct skills and training for the job? Who does what? | Who’s doing what, and why (capture differences in people’s experiences of implementation, what’s important for them etc) |
| Contextual integration, *This refers to the resource work - managing a set of practices through the allocation of different kinds of resources and the execution of protocols, policies and procedures.* | IS IMPLEMENTING THE LAW CHANCE ADEQUATELY SUPPORTED BY PARTICIPATING ORGANISATIONS? Do local and national resources and policies support the implementation? Who gets what, and how? (resource allocation – any additional resources?) | What do people have or need *(capture experiences and views on what people need in order to deliver opt-out as intended)* |
| **REFLEXIVE MONITORING**  *Reflexive Monitoring is the* ***appraisal work*** *that people do to assess and understand the ways that a new set of practices affect them and others around them.* | How is it working? Appraisal and evaluation - assessment of impact, process of reflection, learning and refinement. Evaluating implementation to promote embedding (how a practice is understood and assessed by actors implicated in it) - WHATS WORKED/WORKING |  |
| Systemization  *participants in any set of practices may seek to determine how effective and useful it is for them and for others, and this involves the work of collecting information in a variety of ways.* | DO PEOPLE GET AND USE INFORMATION ABOUT THE EFFECTS OF THE IMPLEMENTATION OF THE LAW CHANGE? Will stakeholders be able to judge the effectiveness of the intervention? how is information obtained to inform appraisal? Outcome and process data | What’s worked/working well or not across the system, (capture, views, thoughts, experiences) |
| Communal appraisal  *participants work together - sometimes in formal collaboratives, sometimes in informal groups to evaluate the worth of a set of practices. They may use many different means to do this drawing on a variety of experiential and systematized information.* | DO PEOPLE COLLECITVELY ASSESS THE LAW CHANGE AS WORTHWHILE? How will stakeholders collectively judge the effectiveness of the intervention? How do participants work together to appraise the intervention? Formal and informal meetings – other | What’s worked/working well or not across the teams/groups, (capture, views, thoughts, experiences) |
| Individual appraisal  *Participants in a new set of practices also work experientially as individuals to appraise its effects on them and the contexts in which they are set. From this work stem actions through which individuals express their personal relationships to new technologies or complex interventions* | DO PEOPE INDIVIDUALLY ASSESS THE LAW CHANGE AS WORTHWHILE? How will individuals judge the effectiveness of the intervention? how do participants evaluate the impact of the intervention individually? Process and context | What’s worked/working well or not for individuals, (capture, views, thoughts, experiences) |
| Reconfiguration  *appraisal work by individuals or groups may lead to attempts to redefine procedures or modify practices - and even to change the shape of a new technology itself.* | DO PEOPLE MODIFY THEIR WORK IN RESPONSE TO APPRAISALS OF THE IMPLEMENTATION OF THE LAW CHANGE? Will stakeholders be able to modify the intervention based on evaluation and experience? can participants modify aspects of the intervention? If so how? | Recommendations: What people recommend for the future. |
